# Meta-Type Analysis of Dopaminergic Effects on Gene Expression in the Neuroendocrine Brain of Female Goldfish

**DOI:** 10.3389/fendo.2012.00130

**Published:** 2012-11-02

**Authors:** Jason T. Popesku, Christopher J. Martyniuk, Vance L. Trudeau

**Affiliations:** ^1^Centre for Advanced Research in Environmental Genomics, Department of Biology, University of OttawaOttawa, ON, Canada; ^2^Canadian Rivers Institute and Department of Biology, University of New BrunswickSaint John, NB, Canada

**Keywords:** dopamine, sub-network enrichment analysis, neurodegeneration, reproduction, immune response

## Abstract

Dopamine (DA) is a major neurotransmitter important for neuroendocrine control and recent studies have described genomic signaling pathways activated and inhibited by DA agonists and antagonists in the goldfish brain. Here we perform a meta-type analysis using microarray datasets from experiments conducted with female goldfish to characterize the gene expression responses that underlie dopaminergic signaling. Sexually mature, pre-spawning [gonadosomatic index (GSI) = 4.5 ± 1.3%] or sexually regressing (GSI = 3 ± 0.4%) female goldfish (15–40 g) injected intraperitoneally with either SKF 38393, LY 171555, SCH 23390, sulpiride, or a combination of 1-methyl-4-phenyl-1,2,3,6-tetrahydropyridine and α-methyl-*p*-tyrosine. Microarray meta-type analysis identified 268 genes in the telencephalon and hypothalamus as having reciprocal (i.e., opposite between agonism and antagonism/depletion) fold change responses, suggesting that these transcripts are likely targets for DA-mediated regulation. Noteworthy genes included ependymin, vimentin, and aromatase, genes that support the significance of DA in neuronal plasticity and tissue remodeling. Sub-network enrichment analysis (SNEA) was used to identify common gene regulators and binding proteins associated with the differentially expressed genes mediated by DA. SNEA analysis identified gene expression targets that were related to three major categories that included cell signaling (STAT3, SP1, SMAD, Jun/Fos), immune response (IL-6, IL-1β, TNFs, cytokine, NF-κB), and cell proliferation and growth (IGF1, TGFβ1). These gene networks are also known to be associated with neurodegenerative disorders such as Parkinsons’ disease, well-known to be associated with loss of dopaminergic neurons. This study identifies genes and networks that underlie DA signaling in the vertebrate CNS and provides targets that may be key neuroendocrine regulators. The results provide a foundation for future work on dopaminergic regulation of gene expression in fish model systems.

## Introduction

Dopamine (DA) is a neurotransmitter important in disorders such as schizophrenia (Seeman and Kapur, 2000) and Parkinson’s disease (Baik et al., 1995), but is also the major neurotransmitter controlling teleost reproduction (reviewed in Dufour et al., 2005; Dufour et al., 2010). In this regard, DA inhibits the release of luteinizing hormone (LH) in fish through multiple mechanisms: (a) DA inhibits gonadotropin-releasing hormone (GnRH) release from GnRH neurons through the D1 receptor (Yu and Peter, 1992); (b) DA directly inhibits LH release from gonadotrophs in the anterior pituitary through the D2 receptor (Peter et al., 1986; Omeljaniuk et al., 1987); (c) DA decreases the expression of GnRH receptor mRNA in the pituitary (Kumakura et al., 2003; Levavi-Sivan et al., 2004); and (d) DA inhibits the synthesis of GABA (Hibbert et al., 2004, 2005), an important stimulator of LH release (Martyniuk et al., 2007). Furthermore, it is well understood that DA, acting through the D1, stimulates growth hormone in fish (Wong et al., 1992). Our recent studies using goldfish have investigated the effects of DA agonists on the hypothalamic transcriptome and proteome (Popesku et al., 2010) or of DA antagonists on gene expression in the neuroendocrine brain (Popesku et al., 2011a). Additionally, we have previously described the effects of a combination of 1-methyl-4-phenyl-1,2,3,6-tetrahydropyridine (MPTP; a selective DA neurotoxin) and α-methyl-*p*-tyrosine (αMPT; a tyrosine hydroxylase inhibitor) on the goldfish hypothalamic transcriptome (Popesku et al., 2008). Using microarray datasets from two of these experiments, and an additional novel microarray data presented here, we further elucidate the mechanistic effects of DA on gene expression in the neuroendocrine brain by performing a meta-type analysis of these datasets.

In transcriptomics, there are a number of bioinformatics approaches to globally assess gene expression data and to organize expression data into a larger biological context. These methods include Gene Ontology (GO) characterization, functional enrichment, and pathway analysis. Many of these approaches have been successfully performed using genomic data in neuroendocrine regions of teleost fishes to better describe cellular events that are mediated by neurotransmitters, hormones, or exogenous neuroactive agents (Marlatt et al., 2008; Popesku et al., 2008; Zhang et al., 2009a; Martyniuk et al., 2010). New bioinformatics tools are now available to construct gene networks using gene expression profiling and have been used successfully in teleost fish (e.g., reverse engineering of adverse pathways for ecotoxicology (Perkins et al., 2011). Sub-network enrichment analysis (SNEA; Ariadne’s Pathway Studio v7.0 Sivachenko et al., 2007) offers a unique approach to protein interaction networks that are described in the literature as well as a curated mammalian database. Specifically, SNEA builds sub-networks by mapping experimental data onto known bio-molecular interactions. The interactions include promoter-binding, protein modification, and common targets of expression. This algorithm has been used to identify gene sub-networks in breast cancer cell lines (Chuang et al., 2007) and is a useful tool for identifying interaction or signaling networks that involve differentially expressed genes. As such, this method can provide insight in gene regulatory pathways.

In this study, we identify genes and sub-networks that are likely regulated by DA based on their reciprocal response to DA agonism or antagonism/depletion. These data have implications for our understanding of DA action in fish neuroendocrine systems.

## Materials and Methods

This is a meta-type analysis of published experiments involving treatments of goldfish with DA agonists (Popesku et al., 2010), antagonists (Popesku et al., 2011a), and after pharmacological depletion of DA (Popesku et al., 2008). The abbreviated Materials and Methods pertaining to the experiments are included here for completeness. It should be noted that, while published, the previous DA depletion studies offered only a cursory analysis of the microarray data in the context of neurotransmitter effects on gene expression and did not specifically address global dopaminergic control of transcriptional responses. Furthermore, we present novel transcriptomic data for specific DA antagonism for which the physiological response to these antagonists has been published (Popesku et al., 2011a), but for which microarray analysis was not performed at that time. We used this novel dataset to compare these DA antagonism responses to agonist and DA depletion responses to improve identification of DA-regulated transcripts in the hypothalamus.

### Experimental animals and conditions

All procedures used were approved by the University of Ottawa Protocol Review Committee and followed standard Canadian Council on Animal Care guidelines on the use of animals in research.

Common adult female goldfish were purchased from a commercial supplier (Aleong’s International Inc., Mississauga, ON, Canada) and maintained at 18°C under a natural simulated photoperiod on standard flaked goldfish food. Fish were allowed to acclimate for a minimum of 1 month prior to any experimental manipulations. Goldfish were anesthetized using 3-aminobenzoic acid ethylester (MS222) for all handling, injection, and dissection procedures.

### Dopamine agonist experiment

Sexually mature, pre-spawning [mid-May; gonadosomatic index (GSI) = 4.5 ± 1.3%] female goldfish (15–40 g) were injected intraperitoneally with either SKF 38393 [D1 agonist; SKF; 1-phenyl-2,3,4,5-tetrahydro-(1H)-3-benzazepine-7,8-diol] or LY 171555 [D2 agonist; LY; (−)-Quinpirole hydrochloride] purchased from Tocris (Ballwin, MO, USA). The experimental design and doses chosen were based on Otto et al. (1999) who showed rapid effects on goldfish brain somatostatin mRNAs. LY was dissolved in physiological saline (0.6% NaCl) to yield a dose of 2 μg/g body weight of fish. SKF was first dissolved in a minimal amount of dimethylsulfoxide (DMSO), and subsequently diluted to 40 μg/g body weight of fish with physiological saline (0.6% for fish). The final concentration of DMSO was 0.099%; DMSO up to 0.1% does not affect basal GH or LH levels (Otto et al., 1999). While 0.1% DMSO may (Mortensen and Arukwe, 2006) or may not (Nishimura et al., 2008) affect gene expression, all of our gene expression work is relative to control fish which received an equivalent amount of DMSO. The fish received two sequential i.p. injections at 5 μL/g body weight each according to the schedule shown in Table 1. The experiment was conducted this way to ensure that all fish received an equivalent volume of vehicle.

**Table 1:** **Injection schedule for the administration of dopamine agonists used in this study**.

Treatment	i.p. Injection 1	i.p. Injection 2	# Fish injected
Control	0.1% DMSO/saline	0.6% Saline	13
SKF	SKF 38393 40 μg/g	0.6% Saline	14
LY	0.1% DMSO/saline	LY 171555 2 μg/g	11

### Dopamine antagonist experiment

The DA D1-specific antagonist SCH 23390 and DA D2-specific antagonist sulpiride were purchased from Tocris (Ballwin, MO, USA). The antagonists were first dissolved in a minimal amount of DMSO, and subsequently diluted with 0.6% saline. The final concentration of DMSO was 0.099%. Sexually regressing (June; GSI = 3 ± 0.4%; *n* = 18 each) female goldfish received a single injection at 5 μL/g body weight of either SCH 23390 or sulpiride to give a dose of 40 μg/g or 2 μg/g body weight of fish, respectively, or saline containing an equivalent amount of DMSO.

### Dopamine depletion experiment

1-Methyl-4-phenyl-1,2,3,6-tetrahydropyridine and α-methyl-*p*-tyrosine (αMPT) were purchased from Sigma-Aldrich (St. Louis, MO, USA). Sexually mature (May; GSI = 4.7 ± 0.6%) female goldfish (*n* = 5 each) were injected with MPTP (50 μg/g; day 0) and αMPT (240 μg/g; day 5) or saline (control) in order to severely deplete catecholamines. Our previous work had established effective doses of MPTP and αMPT in goldfish (Trudeau et al., 1993; Hibbert et al., 2004).

### Tissue dissections

Fish were sacrificed by spinal transection and hypothalami and telencephali tissues were rapidly dissected and immediately frozen on dry ice. Brain tissues were pooled (2–3 hypothalami or telencephali/tube) to increase RNA yield prior to RNA isolation. For the agonists and antagonists, tissues were harvested 5 h post-injection, and for the DA depletion experiment, tissues were harvested 20 h after the αMPT injection. The cerebellae of the fish from the DA depletion experiment were also harvested for brain catecholamine levels, but were not used in further analyses.

### RNA isolation, quantification, and quality assessment

RNA was isolated with the TRIzol method (Invitrogen, Burlington, ON, Canada) per the manufacturer’s protocol. Samples were treated with DNase on-column in an RNeasy Mini Plus kit (Qiagen, Mississauga, ON, Canada). RNA quantity was evaluated using the NanoDrop ND-1000 spectrophotometer (Thermo Fisher Scientific). RNA integrity was evaluated using the BioAnalyzer (Agilent); RIN for each sample was >8.4.

### HPLC analysis of brain catecholamine levels in the dopamine depletion experiment

Catecholamine levels in brain tissues were determined on alumina-extracted samples (100 μL) using HPLC with electrochemical detection (Woodward, 1982). The HPLC incorporated a Varian ProStar 410 solvent delivery system (Varian Chromatography Systems, Walnut Creek, CA, USA) coupled to a Princeton Applied Research 400 electrochemical detector (EG & G Instruments, Princeton, NJ, USA). Concentrations were calculated relative to appropriate standards, using 3,4-dihydroxybenzalamine hydrobromide (DHBA) as an internal standard.

### Microarray hybridizations

For all microarray analyses, cDNA was synthesized from 2 μg total RNA according to the Genisphere 3DNA Array 900MPX kit according to the manufacturer’s protocol (Genisphere, Hatfield, PA, USA). We previously described and validated the production and use of our goldfish-carp cDNA microarray (Martyniuk et al., 2006; Marlatt et al., 2008; Mennigen et al., 2008), and a detailed description of the microarray is available (Williams et al., 2008). Four microarray hybridizations were performed for each hypothalamic and telencephalic tissue pool for both D1 and D2 agonists (total of 16 arrays), antagonists (16 arrays), or DA depletion (MPTP + αMPT; eight arrays) to screen for the effects of the DA in the neuroendocrine brain. For each experiment, three separate pools of RNA from treated fish were hybridized to the microarrays, and a fourth hybridization was a replicate dye-reversal of one of the three RNA pooled samples. Hybridizations were carried out relative to a common pool of control samples (∼30 control fish) for each tissue, which decreases technical variation as only one reference is utilized while maintaining biological variation of the treatment samples (Churchill, 2002). All cDNA synthesis, labeling, and hybridizations were performed using the Genisphere 3DNA Array 900MPX kit according to the manufacturer’s protocol (Genisphere, Hatfield, PA, USA). Hybridizations and scanning protocols were described previously (Martyniuk et al., 2006; Marlatt et al., 2008; Mennigen et al., 2008). Briefly, microarrays were scanned at full-speed 10-μm resolution with the ScanArray 5000 XL system (Packard Biosciences/PerkinElmer, Woodbridge, ON, Canada) using both red and blue lasers. Images were obtained with ScanArray Express software using automatic calibration sensitivity varying photomultiplier (PMT) gain (PMT starting at 65% for Cy5 and 70% for Cy3) with fixed laser power at 80% and the target intensity set for 90%. Microarray images were analyzed with QuantArray (Packard Biosciences/Perkin Elmer), and raw signal intensity values were obtained for duplicate spots of genes. Raw intensity values for all microarray data and microarray platform information have been deposited in the NCBI Gene Expression Omnibus database and assigned the following SuperSeries accession numbers: GSE15855 (agonists), GSE15763 (antagonists), and GSE16044 (MPTP + αMPT). Generalized Procrustes Analysis (Xiong et al., 2008) was used for normalization of the array data and the Significance Analysis of Microarrays (SAM) method (Woodward, 1982; Tusher et al., 2001) was used to identify differentially expressed genes. Genes/ESTs were selected based on identical AURATUS GeneIDs and on the basis of differential regulation in opposite directions for MPTP or the antagonists vs. agonists, or in the same direction for MPTP vs. antagonists; genes that did not fall into one of these categories were not included in the analysis. All genes/ESTs identified and presented were statistically significant (*q* < 5%) in all treatments.

### Real-time PCR

Primers used in this study for aromatase B, 18S, and β-actin have been validated and published (Martyniuk et al., 2006). The Mx3005 Multiplex Quantitative PCR System (Stratagene, La Jolla, CA, USA) was used to amplify and detect the transcripts of interest. Each PCR reaction contained the following final concentrations: 25 ng first strand cDNA template, 1 ×  QPCR buffer, 3 mM MgCl2, 300 nM each F & R primers, 0.25 ×  SYBRGreen (Invitrogen), 200 μM dNTPs, 1.25 U HotStarTaq (Invitrogen), and 100 nM ROX reference dye, in a 25 μL reaction volume. The thermal cycling parameters were an initial one cycle Taq activation at 95°C for 10 min, followed by 40 cycles of 95°C for 30 s, 59°C for 45 s, and 72°C for 30 s. After the reaction was complete, a dissociation curve was produced starting from 55°C (+1°C/30 s) to 95°C. Dilutions of cDNA (1:10–1:31,250) from all samples were used to construct a relative standard curve for each primer set, relating initial template copy number to fluorescence and amplification cycle. For each PCR reaction, negative controls were also introduced including a no-template control (NTC) where RNase-free water was added to the reaction instead of the template (cDNA) and NoRT control, where water was added instead of reverse transcriptase during cDNA synthesis. The SYBR green assay for each target gene was optimized for primer concentration and annealing temperature to obtain, for the standard curve, an *R*^2^ > 0.99, amplification efficiency between 90 and 110% and a single sequence-specific peak in the dissociation curve. No amplification was observed in the NoRT or NTC controls indicating no genomic or reagent contamination. Data were analyzed with the MxPro v4.01 software package.

### Sub-network enrichment analysis of reciprocally DA-regulated transcripts

Pathway Studio 7.1 (Ariadne, Rockville, MD, USA) and ResNet 7.0 were used for SNEA for genes that showed reciprocal expression with MPTP-mediated DA depletion and with the DA agonist SKF 38393. We selected the agonist and DA depletion datasets from the hypothalamus for this analysis because (1) the experiments were conducted at the same time of year (May) and (2) these experiments resulted in the greatest number of reciprocal gene expression changes. A total of 114 genes were successfully mapped to human homologs using the GenBank protein ID while 14 genes could not be confidently mapped to human homologs; hence the unmapped proteins were not included in the analysis. SNEA for expression targets, binding partners, and post-translation modification targets was performed to determine if there were common gene targets for MPTP and SKF treatments. SNEA creates a central “seed” from all relevant entities in the database, to find common effectors (expression targets, binding partners, and post-translational targets). The enrichment *p*-value for gene seeds was set at *p* < 0.05 and, for the current study, the criteria of greater than five members per group were required for inclusion as a significantly regulated gene network. This was chosen to focus the analysis and discussion on the most likely gene networks regulated through DA signaling.

## Results

### Catecholamine depletion

**Figure 1:**
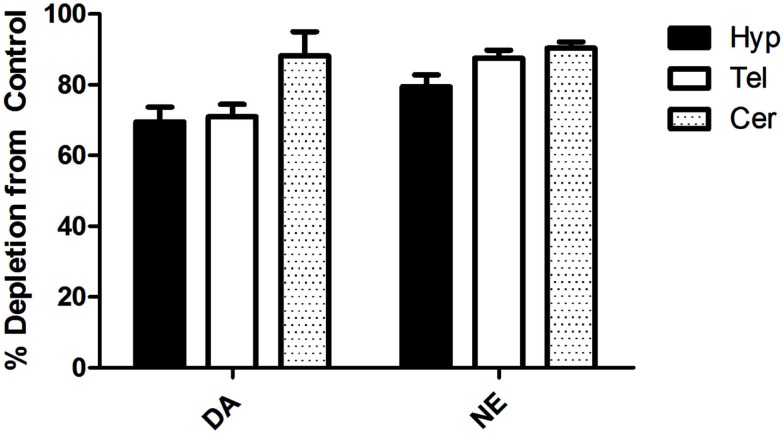
**Percent depletion of dopamine (DA) and norepinephrine (NE) in different brain tissues relative to saline-injected control 6d post-injection with MPTP and 1d post-injection with αMPT (*p* < 0.01 in all cases, relative to control)**. Tel, Telencephalon; Hyp, Hypothalamus; Cer, Cerebellum.

To ensure that the MPTP + αMPT treatment effectively decreased DA levels in the brain, Hyp, Tel, and cerebellum (Cer) tissues were analyzed for catecholamine content using HPLC. Following injections of MPTP (−6 days) and αMPT (−1 day), DA levels were decreased by 69.6 and 70.9% in the Hyp and Tel, respectively, and by 88.2% in the Cer relative to saline-injected controls (Figure 1). Norepinephrine (NE) levels were also reduced in the Hyp (79.4%), Tel (87.5%), and Cer (90.4%).

### Microarray analysis

**Figure 2:**
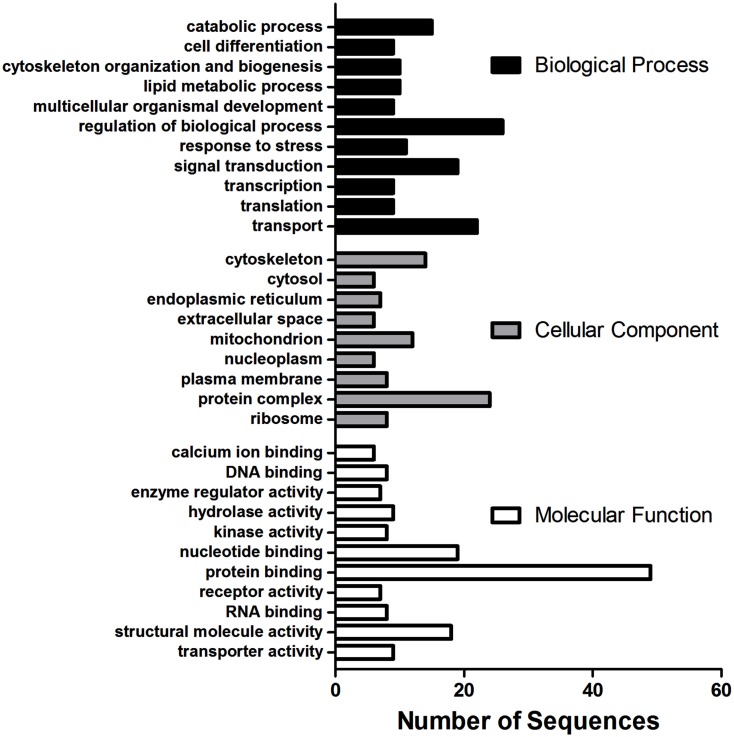
**Multilevel Gene Ontology categorization of 110 annotated ESTs regulated by dopamine in the hypothalamus into their corresponding Biological Process, Cellular Component, and Molecular Function terms**. GO Annotations were first converted to GO-Slim annotations (goslim_generic.obo) and the multilevel chart was constructed using a sequence convergence cutoff of five (seven for Biological Process) to reduce the complexity of the chart.

**Figure A1:**
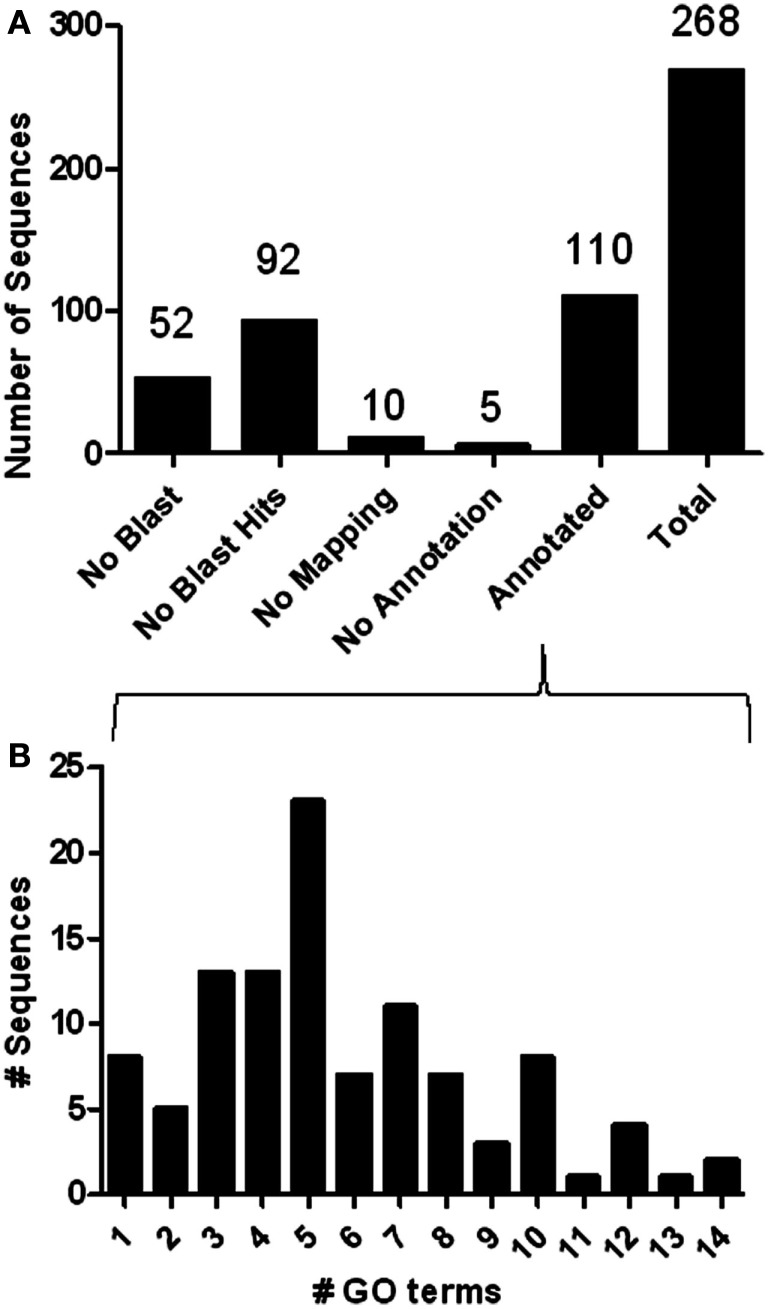
**(A)** Data distribution of the 268 ESTs identified as being differentially regulated (*q* < 5%) by DA in hypothalamus. **(B)** Annotation distribution of the 110 annotated ESTs. In the telencephalon (not shown), a total of four sequences were found of which three had blast hits, and only two were annotated.

Using the microarray datasets from our previous experiments (Popesku et al., 2008, 2010), and the novel microarray data from the antagonist experiment, a meta-type analysis of genes likely regulated by DA was performed. A total of 268 genes/ESTs were identified in the hypothalamus as being regulated by DA, while only four were identified in the telencephalon. Of the 268 genes/ESTs identified in the hypothalamus, only 41% are annotated (Figure A1 in Appendix). The others currently have no known biological function (6%), are not similar to any sequences in GenBank (34%), or are lacking sequence information (19%). The relatively high number of sequences affected by DA in the hypothalamus, the majority of which are acting through the D1 receptor (Table 2), highlights the importance of this receptor in this tissue. The annotated sequences were binned into their corresponding GO Slim terms, using Blast2GO as described in Popesku et al., 2010; Figure 2).

**Table 2:** **Genes/ESTs identified as regulated by dopamine, presented as fold-changes**.

Tissue	AURATUS ID	Best blast hit	Accession	Human homolog		DA depletion or receptor blockage			DA mimic	
				Accession	Gene	MPTP + aMPT	SCH	sulpiride	SKF	LY
Hyp	08j13	14 kDa apolipoprotein	CF662566	No homolog		−1.5			1.7	
Hyp	08b22	17-Beta hydroxysteroid dehydrogenase type 12B, 3-ketoacyl-CoA reductase type B	CA968619	NM_016142	HSD17B12	1.4			−1.7	
Hyp	16j14	26s Protease regulatory subunit 4	CA966407	NM_002802	PSMC1	−1.4			1.4	
Hyp	08e14	40S Ribosomal protein S27	CA968660	NM_001030	RPS27	−1.5			1.7	
Hyp	07f01	Abhydrolase domain containing 12	CA967283	NM_001042472	ABHD12	−1.6			1.8	
Hyp	22n08	Adenylate kinase 3-like 1	CA969490	NM_016282	AK3	1.3			−1.5	
Hyp	08k20	Aldehyde dehydrogenase 7 family, member A1	CA968758	NM_001182	ALDH7A1	−1.3				1.3
Hyp	03h23	Aldolase C	DY231930	NM_005165	ALDOC	1.4			−1.6	
Hyp	05f06	Alpha-2-macroglobulin-1	CF662428	NM_000014	A2M	−1.6			1.5	2.1
Hyp	22i24	Alpha-actin	CA969403	NM_001100	ACTA1	1.4			−1.5	
Hyp	09p02	Angiotensinogen	CA964907	NM_000029	AGT	−1.5			1.8	1.3
Hyp	09j02	Apolipoprotein a-iv	CA966743	NM_000482	APOA4	−1.5			1.7	
Hyp	16n14	Apolipoprotein e	CF662778	NM_000041	APOE	−1.3			2.4	
Hyp	04a17	Aromatase b	FG392770	NM_000103	CYP19A1	1.3			−1.7	
Hyp	14k14	arp2 Actin-related protein 2 homolog	CA964468	NM_005722	ACTR2	−1.3			2.3	1.3
Hyp	12l13	asf1 Anti-silencing function 1 homolog b (*cerevisiae*)	CA966040	NM_018154	ASF1B	−1.3			1.9	
Hyp	16l15	atp-Binding sub-family f member 2	CA966450	NM_007189	ABCF2	−1.3			1.6	
Hyp	16o14	BC-10 protein	CA966992	NM_006698	BLCAP	−1.3			1.9	
Hyp	03o22	Beta-actin	DY232011	NM_001101	ACTB	1.3			−1.6	
Hyp	22l24	Branched chain ketoacid dehydrogenase kinase	CA969461	NM_005881	BCKDK		1.6		−1.8	
Hyp	02a23	Calmodulin 1b	FG392553	no homolog		1.2			−1.7	
Hyp	14g01	Claudin 23	CA964745	NM_194284	CLDN23	−1.4			1.8	
Hyp	14k02	Coiled-coil domain containing 47	CA964457	NM_020198	CCDC47	−1.3			2.1	
Hyp	19a04	Cold shock domain-containing protein e1	CA964993	NM_001007553	CSDE1		−1.4		1.5	1.3
Hyp	08o15	Complement C3-H2	CA970421	NM_000064	C3	−1.4			1.6	
Hyp	08b20	Complement component q subcomponent-like 4	CA968617	NM_001008223	C1QL4	−1.3				1.3
Hyp	02c23	Creatine kinase b variant 1	DY231608	NM_001823	CKB	1.3			−1.6	
Hyp	02n10	Creatine testis isozyme	DY231690	NM_001824	CKM	1.2			−1.5	
Hyp	21l19	C-type lectin	CA969207	no homolog		1.5	−1.7		−1.7	
Hyp	19a14	Cubilin (intrinsic factor-cobalamin receptor)	CA964997	NM_001081	CUBN	−1.4			1.4	
Hyp	17g09	Cxxc finger 1 (phd domain)	CA964951	NM_001101654	CXXC1	1.3			−1.7	
Hyp	06d13	Cytochrome P450 2F2-like	CA965416	NM_007817	CYP2F2	−1.4				1.6
Hyp	05l01	Cytokine induced apoptosis inhibitor 1	CA966987	NM_020313	CIAPIN1	−1.4			2.3	
Hyp	03f23	Deoxyribonuclease I-like 3	DY231911	NM_004944	DNASE1L3	1.5			−1.5	
Hyp	23k24	e3 Ubiquitin protein ligase	CA968074	NM_007013	WWP1	1.6			−1.6	
Hyp	02i24	Ependymin	DY231713	NM_017549	EPDR1	1.3			−1.6	
Hyp	03o21	Ependymin	DY232010	NM_017549	EPDR1	1.4			−1.7	
Hyp	24a12	eph Receptor a7	CA969719	NM_004440	EPHA7	1.6			−2.1	
Hyp	15a10	Equilibrative nucleoside transporter 1	CA965545	NM_001078174	SLC29A1	1.3			−1.6	
Hyp	07b01	Eukaryotic translation elongation factor-1 gamma	CA966738	NM_001404	EEF1G	−1.5			1.7	
Hyp	20j14	Eukaryotic translation initiation factor 2, subunit 1 alpha	CA966561	NM_004094	EIF2S1	−1.3	−2.0		2.3	
Hyp	09e01	Fibronectin 1b	CA964120	NM_212482	FN1	−1.3			2.0	1.3
Hyp	24j21	fk506-Binding protein 1a	CA966789	NM_054014	FKBP1A	1.3			−1.5	
Hyp	03o09	Fructose-bisphosphate aldolase c	FG392624	NM_005165	ALDOC	1.4			−1.6	
Hyp	10m11	g Protein-coupled family group member c	CA967701	NM_024051	GGCT	1.3			−1.6	
Hyp	17n11	Gamma-glutamyl cyclotransferase	CA965786	NM_024051	GGCT	1.3			−1.7	
Hyp	03i20	Glutamine synthetase	DY231974	NM_001033044	GLUL	1.2			−1.5	
Hyp	10d04	Glutathione peroxidase 3	CA964192	NM_002084	GPX3	1.4			−1.5	
Hyp	23o12	Glyceraldehyde 3-phosphate dehydrogenase	CA968103	NM_002046	GAPDH	2.0			−2.1	
Hyp	08h01	Glyceronephosphate-*O*-acyltransferase	CA968696	NM_014236	GNPAT	−1.6			2.2	
Hyp	14b13	Granulin 1	CA964295	NM_002087	GRN	−1.3			1.5	
Hyp	19m14	h2a Histone member y2	CA965061	NM_018649	H2AFY2	−1.4			1.6	
Hyp	14k03	Heat shock protein 90 beta	CA964458	NM_007355	HSP90AB1	−1.3			1.7	
Hyp	14i04	HECT domain containing 1	CA964417	NM_015382	HECTD1		−1.4		1.5	
Hyp	24o12	Hexokinase I	CA969997	NM_000188	HK1	1.6			−1.9	
Hyp	08g14	High-density lipoprotein binding protein	CA968690	NM_005336	HDLBP	−1.4			1.6	
Hyp	19d02	Hydroxysteroid (17-beta) dehydrogenase 10	CA965806	NM_001037811	HSD17B10	−1.3			2.2	1.3
Hyp	03i10	Immunoglobulin mu heavy chain	FG392590	XM_003120441	LOC100510678	1.5			−1.5	
Hyp	04j23	Jumonji domain containing 3	FG392963	NM_001080424	KDM6B	1.3			−1.5	
Hyp	13o14	Latexin	CF662717	NM_020169	LXN		−1.7		1.6	
Hyp	22g07	Leucine-rich repeat (in flii) interacting protein 1	CA969350	NM_001137550	LRRFIP1	1.2			−1.7	
Hyp	11p01	Leucine-rich repeat containing 58	CF662658	NM_001099678	LRRC58	−1.3			2.2	
Hyp	19f13	Loc548392 protein	CA969104	unknown		−1.4			2.0	
Hyp	14m01	Malate dehydrogenase 1, NAD (soluble)	CA964750	NM_005917	MDH1	−1.3			1.8	1.3
Hyp	12k14	Male-specific protein	CA970272	NM_001012241	MSL1	−1.3			1.9	
Hyp	22o11	Map microtubule affinity-regulating kinase 4	CA969512	NM_031417	MARK4	1.5			−2.0	
Hyp	21l16	Membrane palmitoylated	CA966525	NM_002436	MPP1	1.9			−1.6	1.3
Hyp	09p22	Methylcrotonoyl-coenzyme a carboxylase 2	CA964915	NM_022132	MCCC2	1.5			−1.8	
Hyp	22k08	MHC class I antigen	CA969424	unknown		1.4			−2.0	
Hyp	08a03	mid1 Interacting g12-like protein	CA970376	NM_021242	MID1IP1	−1.3			1.6	
Hyp	09k02	mid1 Interacting g12-like protein	CA964854	NM_021242	MID1IP1	−1.4			1.7	
Hyp	08l01	Middle subunit	CA965449	NM_002032	FTH1	−1.4			2.5	
Hyp	03k10	Midkine-related growth factor b	FG392604	no homolog		1.4			−1.5	
Hyp	12n01	Mitochondrial ribosomal protein l19	CA966046	NM_014763	MRPL19	−1.6			1.5	
Hyp	19p16	Mitochondrial ribosomal protein l20	CA967272	NM_017971	MRPL20		−1.4		2.0	
Hyp	11j11	Mitogen-activated protein kinase 7 interacting protein 3	CF662634	NM_003188	MAP3K7	1.4			−1.7	
Hyp	12p13	m-Phase phosphoprotein 6	CA966058	NM_005792	MPHOSPH6	−1.5			2.1	
Hyp	06g06	Myelocytomatosis oncogene b	CF662485	NM_002467	MYC	1.3			−2.7	
Hyp	14n02	Myosin regulatory light chain	CA964520	NM_013292	MYLPF	−1.3			1.6	
Hyp	24b19	nck Adaptor protein 2	CA969746	NM_003581	NCK2	1.4			−1.5	
Hyp	19l18	Negative elongation factor d	CA965844	NM_198976	TH1L		−1.8		1.5	
Hyp	03i12	Nel-like protein 2	FG392591	NM_001145107	NELL2	1.3			−1.7	
Hyp	16k15	nlr Card domain containing 3	CF662774	NM_178844	NLRC3		−1.3		1.8	
Hyp	18c18	Nol1 nop2 sun domain member 2	CA964613	NM_017755	NSUN2		−1.5		−1.5	
Hyp	08o01	Novel protein	CA968809	no homolog		−1.4			1.5	
Hyp	11d07	Novel protein	CF662614	no homolog		1.3			−1.5	
Hyp	15i06	Novel protein (zgc:136439)	CA965636	no homolog			−1.6		1.6	
Hyp	15b13	Novel protein lim domain only 3 (rhombotin-like 2; zgc:110149)	CA965552	NM_001001395	LMO3	−1.4			2.0	
Hyp	11e15	Novel sulfotransferase family protein (cytosolic sulfotransferase)	CA965939	NM_001055	SULT1A1	−1.3			1.9	
Hyp	19e01	Nuclear receptor sub-family group member 2	CA966183	NM_005126	NR1D2	−1.4			2.1	
Hyp	15e23	Phosducin-like 3	CA966723	NM_024065	PDCL3		1.5		−1.6	
Hyp	12l11	Plasma retinol-binding protein 1	CA966039	NM_006744	RBP4	1.3			−1.5	
Hyp	03k09	Poplar cDNA sequences	FG392603	no homolog		1.3			−1.5	
Hyp	08g04	Prostaglandin h2 d-isomerase	CA968684	NM_000954	PTGDS	−1.5			1.5	
Hyp	22p03	Proteasome (macropain) 26s non-4	CA969527	NM_002810	PSMD4	1.4			−1.6	
Hyp	12b01	Proteasome (macropain) alpha 5	CA965983	NM_002790	PSMA5	−1.5			2.0	1.3
Hyp	12i01	Purine nucleoside phosphorylase	CA967769	NM_000270	PNP	−1.5			1.6	
Hyp	22b23	Response gene to complement 32	CA969259	NM_014059	C13orf15	1.3			−2.0	
Hyp	22g21	Ribosomal protein l13	CA969362	NM_000977	RPL13	1.5			−1.7	
Hyp	08o16	Ribosomal protein l27a	CA968817	NM_000990	RPL27A	−1.3			1.5	
Hyp	12d13	Ribosomal protein l27a	CA965998	NM_000990	RPL27A	−1.5			1.6	
Hyp	09o01	Serine incorporator 1	CA964172	NM_020755	SERINC1	−1.4				1.5
Hyp	21a01	sh3-Domain grb2-like 2	CA967895	NM_003025	SH3GL1	−1.5			1.7	1.3
Hyp	09g14	si:ch211-Protein	CA964823	no homolog		−1.4			1.8	
Hyp	24i19	StAR-related lipid transfer (START) domain containing 4	CA969885	NM_139164	STARD4	1.4			−2.1	1.6
Hyp	09n02	Sterol-c5-desaturase (fungal delta-5-desaturase) homolog (*cerevisiae*)	CA964885	NM_006918	SC5DL	−1.3			2.5	
Hyp	12p21	Surfeit 4	CA966062	NM_033161	SURF4		−1.5		1.6	
Hyp	20o02	Tetraspanin 9	CA965906	NM_006675	TSPAN9	−1.6			1.6	
Hyp	24i22	Transaldolase 1	CA969888	NM_006755	TALDO1	1.4			−1.6	
Hyp	15f10	Translocon-associated protein subunit delta precursor	CA965601	NM_006280	SSR4	1.3			−1.8	
Hyp	12f01	Transthyretin precursor	CA966004	NM_000371	TTR	−1.3			3.0	
Hyp	07h01	Triosephosphate isomerase	CA968504	NM_000365	TPI1	−1.3			1.8	
Hyp	14f24	Troponin c-type 2	CA964383	NM_003279	TNNC2	1.4			−2.1	
Hyp	21g17	Troponin c-type 2	CA967929	NM_003279	TNNC2		−1.5		1.6	
Hyp	22g09	Tubulin alpha 8 like 4	CA969352	NM_006082	TUBA1B	1.3			−1.8	
Hyp	03o23	Tubulin beta-2c	FG392672	NM_006088	TUBB2C	1.4			−1.5	
Hyp	17j23	Tubulin beta-2c chain	CA965774	NM_006088	TUBB2C	1.4			−1.5	
Hyp	14f02	u2 Small nuclear RNA auxiliary factor-1	CA964363	NM_006758	U2AF1		−1.7		2.4	1.3
Hyp	22l09	Vacuolar protein sorting 13c	CA969449	NM_018080	VPS13C	1.3			−1.5	
Hyp	20j02	Vacuolar protein sorting 4a	CA966560	NM_013245	VPS4A	−1.5			1.7	1.6
Hyp	14j12	Vimentin	CA964445	NM_003380	VIM	1.4			−1.5	
Hyp	24i24	Vimentin	CA969890	NM_003380	VIM	1.4			−1.7	
Hyp	12i13	Vitellogenin 2	CA967775	no homolog		−1.3			1.4	
Hyp	19o08	Zinc and double phd fingers family 2	CA965067	NM_006268	DPF2		−1.5		1.5	
Hyp	23a24	Zinc finger ccch-type containing 7a	CA967982	NM_017590	ZC3H7B	2.0			−2.3	
Hyp	15i14	Zinc finger protein 782	CA965639	NM_001001662	ZNF782	−1.3			2.0	
Hyp	20c13	Zona pellucida glycoprotein	CA966260	no homolog			−1.6		1.7	
Tel	12o17	ccaat Enhancer-binding protein beta	CA967804	NM_005194	CEBPB			−1.6		1.7
Tel	12e10	Leucine-rich ppr-motif containing	CA970240	NM_133259	LRPPRC	−1.8				1.6
Tel	14f04	Solute carrier family 2 (facilitated glucose fructose transporter) member 5	CA964365	NM_207420	SLC2A7			−1.3		1.9

*ESTs were manually selected based on identical AURATUS GeneIDs and on the basis of differential regulation in opposite directions for MPTP or the antagonists vs. agonists, or in the same direction for MPTP vs. antagonists. All ESTs were identified as being differentially regulated (*q* < 5%) in all treatments. Only those with BLAST hits (NCBI), obtained with Blast2GO, are shown. Duplicate names may exist in the list, but were not identified by sequence overlap (cap3) and may represent separate genes or individual isoforms. The median “minimum ExpectValue” = 1.9E−57 and the average “mean similarity” = 84.8% ± 1%. In the case where a suitable BlastX hit was unavailable, the best BlastN hit is used and is listed in the complete table in the supplemental data (Table A1 in Appendix). SCH, SCH 23390; SKF, SKF 38393; LY, LY 171555*.

### Real-time RT-PCR validation of AromB

**Figure 3:**
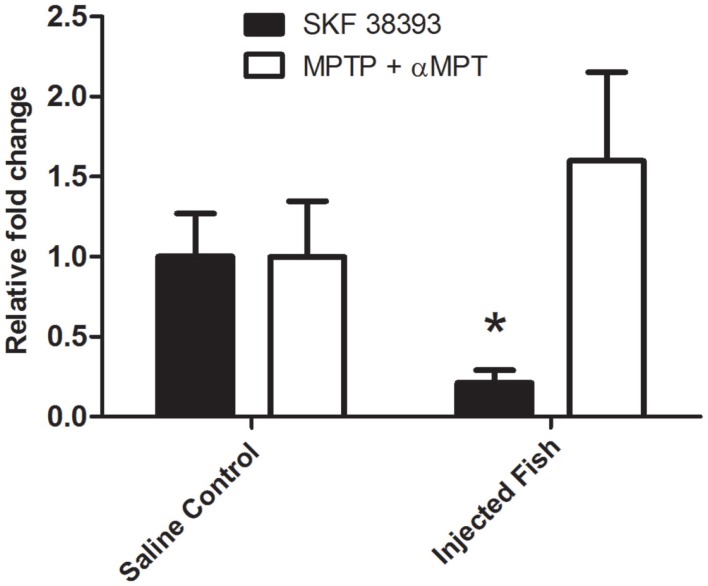
**Real-time RT-PCR of aromatase B mRNA levels in the hypothalamus of SKF 38393-injected fish after 5 h or MPTP + αMPT-injected fish after 24 h. SKF 38393 data were normalized to β-actin and MPTP + αMPT data were normalized to 18S as they were determined to be the most stable for the respective experiments**. A Mann–Whitney U Rank Sum test was performed on injected vs. control fish with significance (*) considered at *p* < 0.05 (two-tailed).

Changes in the hypothalamic mRNA levels of Aromatase B identified by microarray analysis were validated using real-time RT-PCR. Figure 3 shows a 4.7-fold decrease (*p* = 0.027) in AromB mRNA levels 5 h post-injection with SKF 38393. AromB mRNA levels were increased 1.6-fold following DA depletion, but did not reach statistical significance (*p* > 0.05).

### SNEA

**Figure 4:**
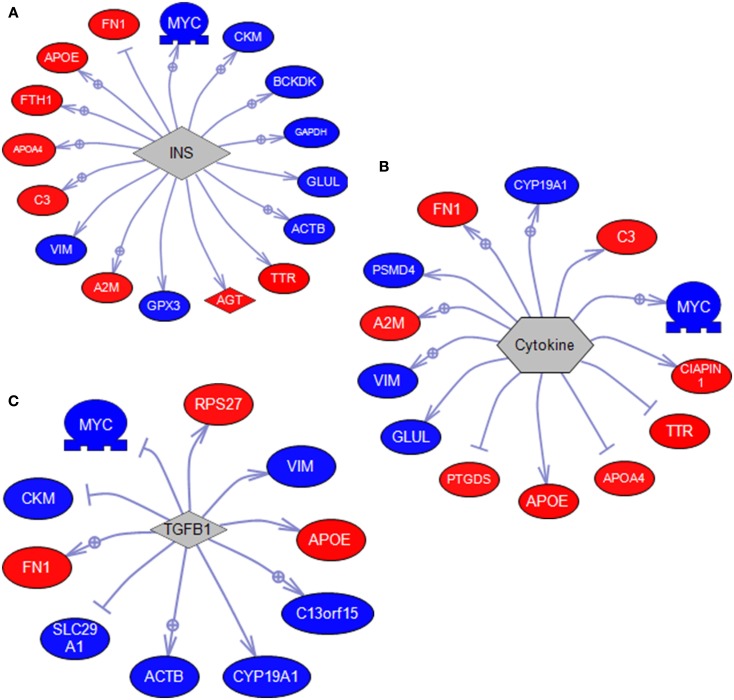
**SNEA diagrams showing the gene set target relationships for (A) insulin, (B) cytokines, and (C) TGFβ1, represented by arrows**. Arrows with a+ in a circle indicate a positive effect in addition to a relationship. Dead-head arrows (–|) indicate a negative effect in addition to a relationship. Directional changes of up (Red) and down (Blue) are color-coded. Results are shown relative to SKF 38393 with changes/color being opposite for MPTP + αMPT. Gene abbreviations are listed in Table 2.

Sub-network enrichment analysis identified a number gene set targets for MPTP-mediated DA depletion and SKF 38393 (Table 3). Expression targets of insulin (INS) were highly affected by DA deletion and receptor stimulation (Figure 4A). This expression group included genes such as *apoe* and *apoa4*, *vim*, *gapdh*, and *myc*. Expression targets also affected by DA depletion and SKF 38393 were those related to cell signaling, for example expression targets of STAT3, SMAD, JUN, and SP1 signaling. A second major group of expression targets included those related to inflammation such as cytokines, NF-κB, IL-6, IL-1β, and TNF. Genes involved in cytokine signaling that are reciprocally affected by dopaminergic stimulation/inhibition included *fn1*, *cyp19a1*, *psmd4*, *vim*, and *glul* (Figure 4B). The third group involved expression targets related to cell growth and differentiation such as insulin-like growth factor I (IGF1) and transforming growth factor-beta (TGFβ1; Figure 4C). Also noteworthy was that expression targets of HIF1A were also identified in the SNEA analysis (Table 3). SNEA is also able to identify binding partner networks and post-translational targets using differentially expressed genes. Binding partners of vitamin D, GAPDH, myosin, and tubulin were affected by treatments while protein modification targets of trypsin and glutathione transferase were significantly impacted through DA signaling (Table 3).

**Table 3:** **Sub-network enrichment analysis groupings of genes identified as being regulated by dopamine**.

Name	Gene set seed	Overlapping entities	*p*-Value
Expression targets	INS	AGT, FN1, MYC, GAPDH, GLUL, GPX3, APOE, TTR, VIM, C3, APOA4, A2M, ACTB, FTH1, CKM, BCKDK	1.37E−06
	STAT3	FN1, MYC, VIM, APOA4, A2M, HSP90AB1, CYP19A1, C13orf15	6.46E−04
	PGR	FN1, MYC, GAPDH, CYP19A1, C13orf15	1.02E−03
	SP1	AGT, FN1, MYC, APOE, VIM, C3, SLC29A1, CYP19A1, SH3GL1, SULT1A1, ASF1B, CKM, BCKDK, CKB, CYP2F1	1.21E−03
	NR3C1	AGT, FN1, MYC, GAPDH, GLUL, CYP19A1, SULT1A1	1.43E−03
	JUN	FN1, MYC, GLUL, APOE, VIM, A2M, CYP19A1, TPI1	1.48E−03
	AKT1	FN1, MYC, GAPDH, MAP3K7, VIM, A2M, CYP19A1, CKM	2.10E−03
	CEBPA	AGT, MYC, GAPDH, GLUL, TTR, C3, APOA4, ACTB	3.63E−03
	SMAD	FN1, MYC, VIM, C13orf15, CKM	3.92E−03
	IGF1	AGT, FN1, MYC, VIM, FKBP1A, CYP19A1, TUBA1B, ACTB	4.86E−03
	SMAD3	FN1, MYC, VIM, CYP19A1, CKM	5.56E−03
	HGF	FN1, MYC, EIF2S1, VIM, C3, A2M	5.59E−03
	SRC	FN1, MYC, A2M, CYP19A1, PSMD4	6.62E−03
	Cytokine	FN1, MYC, PTGDS, GLUL, APOE, TTR, VIM, C3, APOA4, A2M, CYP19A1, CIAPIN1, PSMD4	6.86E−03
	HIF1A	FN1, MYC, GAPDH, VIM, SLC29A1, PSMD4	7.38E−03
	PI3K	FN1, MYC, MAP3K7, FKBP1A, SLC29A1, HSP90AB1, CYP19A1, CKM	8.94E−03
	NF−kB	FN1, MYC, PTGDS, GAPDH, GLUL, GRN, APOE, VIM, C3, A2M, CYP19A1	8.97E−03
	TP53	AGT, FN1, MYC, PTGDS, GAPDH, SLC29A1, HSP90AB1, CKM, PSMD4	9.81E−03
	Jun/Fos	FN1, MYC, PTGDS, APOE, TTR, VIM, A2M, CYP19A1, TPI1	1.12E−02
	STAT	AGT, FN1, MYC, C3, A2M	1.55E−02
	CTNNB1	FN1, MYC, GLUL, VIM, PSMD4	1.67E−02
	PKC	FN1, MYC, PTGDS, GLUL, GRN, APOE, HSP90AB1, CYP19A1	1.69E−02
	IL-6	FN1, MYC, APOE, TTR, A2M, HSP90AB1, CYP19A1, CKM	1.71E−02
	Endotoxin	PTGDS, GAPDH, APOE, A2M, ACTB	2.32E−02
	IL-1β	FN1, PTGDS, VIM, C3, A2M, HSP90AB1, ACTB, FTH1	2.35E−02
	IFNG	AGT, FN1, MYC, GAPDH, APOE, VIM, C3, A2M, HSP90AB1, TUBA1B	2.96E−02
	TNF	AGT, FN1, MYC, PTGDS, GAPDH, GLUL, APOE, VIM, C3, CYP19A1, ACTB	3.75E−02
	EP300	AGT, FN1, GAPDH, HSP90AB1, CKM	4.67E−02
	TGFB1	FN1, MYC, APOE, VIM, SLC29A1, CYP19A1, ACTB, C13orf15, CKM, RPS27	4.86E−02
	LEP	FN1, MYC, GAPDH, APOA4, CYP19A1	4.91E−02
Binding partners	Vitamin D	C3, APOA4, CUBN, ACTA1	2.81E−05
	GAPDH	FN1, GAPDH, FKBP1A, TUBA1B	7.44E−04
	HDL	FN1, TTR, A2M, HDLBP	1.36E−03
	APP	FN1, TTR, A2M, HSD17B10	1.92E−03
	Myosin	GAPDH, VIM, ACTB, MPP1	3.63E−03
	Tubulin	MAP3K7, APOE, TPI1, HK1, LRPPRC, EEF1G	5.21E−03
	ATP	MAP3K7, APOE, HSP90AB1, MCCC2	4.82E−02
Protein modification targets	Trypsin	AGT, FN1, GLUL, VIM, C3, A2M	4.39E−03
	GST	VIM, FKBP1A, TALDO1, NSUN2	8.28E−03

## Discussion

Our approach is an effort to identify a group of genes that are likely regulated by DA. The principle behind the analysis is that genes commonly affected in one direction by severe catecholamine depletion (MPTP + αMPT) and/or DA antagonists will also be affected by DA agonists but expression changes will be in the opposite direction. The power and novelty of this analysis lies in the physiological manipulation and biological validation of reciprocal fold-changes between DA agonists and antagonists/depletion *in vivo*, rather than the technical validation resulting from different techniques performed on the same samples. Additionally, we validated the expression of brain aromatase in the hypothalamus (discussed below) using real-time RT-PCR.

Here we present transcripts that are affected by well-characterized dopaminergic manipulations and allow for speculation on DAergic mechanisms of action in the goldfish neuroendocrine brain. Furthermore, our analysis identified gene networks and provides the foundation for future work on DAergic regulation of neuroendocrine gene expression. Some of the genes/ESTs identified in this analysis (e.g., calmodulin, apolipoprotein) were previously discussed (Popesku et al., 2010) and will not be discussed here. It is not our intention to examine all of the genes/ESTs listed in Table 2, but we have selected some to discuss in terms of current and emerging ideas in dopaminergic neuron (dys)function. The genes/ESTs below are discussed relative to DA receptor stimulation.

The DA agonists and the DA depletion experiments provided the greatest number of reciprocal changes in gene expression compared to the DA antagonist experiment, which is likely due to the fact that both the agonist and depletion experiments were conducted at the same time of year (May) when the fish were of similar sexual maturity (GSI ∼4.6%) compared to the antagonist experiment (June) when fish were sexually regressing (GSI ∼3%). The difference in the number of gene changes between these time points highlights the importance of seasonality of dopaminergic action in the neuroendocrine brain of fish (Zhang et al., 2009b). Indeed, the inhibitory tone of DA on gonadotropin release at these times of year indicate that the fish are in different physiological states (Trudeau et al., 1993; Vacher et al., 2002) and thus may respond to DAergic manipulation differently. This is apparent in some of the genes listed in Table 2 (full list in Table A1 in Appendix), and is a limitation of our approach. We are, however, comparing the effects of DAergic manipulation against paired control fish and are looking for genes that are consistently differentially expressed as a result of that manipulation. While few genes were differentially expressed in the DA antagonist experiment when compared to the other two datasets, the new microarray data presented here provides some further insight into teleost brain function.

Norepinephrine levels were severely reduced in addition to DA levels in MPTP + αMPT-treated fish; however, the genes discussed below are limited to those showing opposite changes to specific DA agonists supporting the hypothesis that genes are therefore likely regulated by DA itself.

The identification of ependymin and vimentin in the hypothalamus highlights the significance of neuronal plasticity and tissue remodeling in response to DAergic manipulations. Ependymin is an extracellular glycoprotein and neurotrophic growth factor involved in optic nerve regeneration, synaptic plasticity, and long-term potentiation in Cypriniformes (Shashoua, 1991; Adams and Shashoua, 1994; Adams et al., 1996). Moreover, ependymin was shown to be overexpressed in regenerating echinoderms (Suarez-Castillo et al., 2004). Ependymin-related proteins were identified in amphibians and mammals (Suarez-Castillo and Garcia-Arraras, 2007) and Shashoua et al. (2001) showed that a short fragment of goldfish ependymin was able to activate the AP-1 transcription factor in neuroblastoma and primary rat brain cortical cultures. Similarly, vimentin is an intermediate filament and is known to increase during cerebellar regeneration in the brown ghost knifefish, *Apteronotus leptorynchus* (Clint and Zupanc, 2002). At least 2 forms of vimentin exist in goldfish (Glasgow et al., 1994), and while the current analysis is unable to resolve the form(s) of vimentin regulated by DA, it is likely that both of the sequences listed in Table 2 correspond to the same form, as they share nearly identical expression patterns in response to DA. Both vimentin and ependymin, along with α- and β-actin and tubulins (Table 2) were decreased in response to DA, supporting the role of DA in synaptic plasticity and tissue remodeling (Kauer and Malenka, 2007). Cytoskeletal remodeling is hypothesized to be important for hormone secretion from the anterior pituitary in mammals (Ravindra and Grosvenor, 1990). Furthermore, Ravindra and Grosvenor (1988) demonstrated that domperidone, a D2-specific antagonist that does not cross the blood-brain barrier but can act on the pituitary, increased prolactin (PRL) levels as well as pituitary polymerized tubulin levels, similar to levels seen in suckling rats. This response, the authors observed, was blocked by bromocriptine, a D2-specific agonist supporting a role for DA in changes observed in the tubulin system in the anterior pituitary. This is relevant because, in fish, it should be noted that DAergic neurons in the mediobasal hypothalamus (e.g., posterior tuberculum) project directly to the pituitary (i.e., are hypophysiotropic; Hornby and Piekut, 1990; Anglade et al., 1993). This is important as it suggests the need for maintaining DA neuronal populations throughout seasonal reproductive period. The identification of aromatase b (CYP19B, or AroB) in our analysis as being inhibited by DA is of particular interest. Our RT-PCR targeted validation of the decrease in AroB mRNA levels in response to SKF 38393, it also confirmed an opposite change in direction of AroB mRNA levels in response to DA depletion as identified by the microarray. In adult fish, AroB is expressed only in radial glial cells (Diotel et al., 2010; Le Page et al., 2010), which persist throughout life and serve as neuronal progenitors in the brain. At least some AroB-immunoreactive (ir) neurons in the medial preoptic area (POA) of the Japanese quail brain respond to DA (Cornil et al., 2004) and a few AroB-ir neurons in the POA of the bluehead wrasse are in close proximity with, while a subset appear to co-express, tyrosine hydrolase (TH; Marsh et al., 2006), the rate-limiting step in DA synthesis and a marker for cathecholaminergic neurons. Moreover, some TH-ir neurons in the POA of rainbow trout express estrogen receptors (Linard et al., 1996) and testosterone and estradiol increase goldfish pituitary DA turnover rates as measured following αMPT-induced catecholamine depletion (Trudeau et al., 1993). More importantly, DA was shown to reduce aromatase enzyme activity in quail POA homogenates *in vitro* (Baillien and Balthazart, 1997). These studies, including the current one, suggest that DA regulates AroB, possibly to modulate the feedback mechanisms of sex steroids on the brain. However, AroB is also important in neurogenesis and brain repair (reviewed in Diotel et al., 2010). Interestingly, Pollard et al. (1992) showed full recovery of DA levels in the brain of goldfish after 8 days using a moderate dose of MPTP (50 μg/g), and Poli et al. (1992) demonstrated spontaneous recovery of DA and NE levels in the goldfish telencephalon, diencephalon, and medulla after 6 weeks following injection of MPTP at a lower dose (10 μg/g) for three consecutive days. These two studies suggest that in fish, unlike in mammals, DA neurons regenerate following injection with MPTP, and may be linked to higher aromatase activity in the fish brain. This is an avenue of research we are currently conducting.

Multiple genes/ESTs identified as being regulated by DA are involved in the lipid and fatty acid metabolic process or transport. For example, 17β-hydroxysteroid dehydrogenase type 12B (HSD17B12; down), high-density lipoprotein binding protein (HDLBP; up), vitellogenin 2 (vtg2; up), cubulin (CUBN; up), sh3-domain grb-like 2 (SH3GL1; up), StAR-related lipid transfer domain containing 4 (STARD4; down), and sterol-c5-desaturase homolog (SC5DL; up) were identified as being regulated by DA. SC5D is involved in the biosynthesis of cholesterol (Sugawara et al., 2001). HSD17B12 reduces 3-ketoacyl-CoA to 3-hydroxyacyl-CoA in the second step of fatty acid elongation (Moon and Horton, 2003). *In vivo* studies in zebrafish demonstrated that HDLBP is not affected by the insulin family or growth hormone, but it is hypothesized that HDLBP is involved in lipid transfer based on its high expression in the liver and ovary (Chen et al., 2003). CUBN is a high-density lipoprotein receptor (Moestrup and Kozyraki, 2000) and STARD4 is hypothesized to facilitate transport of a cholesterol precursor (Soccio et al., 2002). Vtg is best characterized as a liver phosphoprotein stimulated by estrogen and then deposited in the ovary (Jalabert, 2005; Kang et al., 2007), but is, in general, a lipid transport molecule. The changes in these mRNAs suggest lipid mobilization, possibly to derive energy for neuronal remodeling as discussed above.

The granulins are conserved growth factors and are able to stimulate the proliferation of macrophages in goldfish (Hanington et al., 2006). Granulin also has protease inhibitor activity in invertebrates (Hong and Kang, 1999) and cysteine protease activity in plants (Chen et al., 2006). Granulin was shown to be relatively lowly expressed in the brain of goldfish (Hanington et al., 2006) and tilapia (Chen et al., 2007). It appears as though DA, acting through the D1 receptor, stimulates expression of granulin in the hypothalamus of female goldfish. In the developing rat hypothalamus, it was demonstrated that both estrogen and androgen induced granulin expression (Suzuki et al., 2001) and that estrogen induced granulin expression in the dentate gyrus (hippocampus) of adult rats (Chiba et al., 2007). Furthermore, in hippocampal rat tissue *in vitro*, estradiol enhanced neural progenitor cell proliferation and this response was blocked by a granulin-specific antibody (Chiba et al., 2007). Although speculative, this is relevant, as hydroxysteroid (17β) dehydrogenase was identified here as being increased in response to DA, which interconverts 17β-estradiol and estrone, 16-α-hydroxyestrone and estriol, and androstenedione and testosterone Stoffel-Wagner (2003), suggesting that sex steroids influence the DAergic regulation of granulin or, alternatively, the DA modulates estrogen-regulated granulin expression.

Granulin mRNA levels were also identified as being decreased 4.2-fold in the goldfish telencephalon following a 2-days waterborne exposure to 0.1 μM thyroid hormone (T3; Wiens, 2009). While unconfirmed, this is intriguing because the current study identified transthyretin (TTR) mRNA levels as being significantly increased in response to DA. TTR is a thyroid hormone-binding and transport protein and is necessary for maintaining normal levels of circulating thyroid hormone in plasma (Episkopou et al., 1993). Furthermore, TTR protein levels are increased in the cerebrospinal fluid (CSF) of rats with degenerating nigrostriatal neurons (Rite et al., 2007). Future studies aimed at examining the potential interaction between T3 and DA are warranted, particularly as microarray analysis identified increases in mRNA levels of iodothyronine deiodinase type I in the hypothalamus of female fish in response to SKF 38393 and 171555 (D1- and D2-specific agonists, respectively; Popesku et al., 2010).

The identification of U2 small nuclear RNA auxiliary factor-1 (U2AF1) mRNA levels as being increased by DA acting through the D1 receptor (Table 2) is interesting. There are currently five known small nuclear ribonucleoproteins (snRNPs) that make up the spliceosome (Query, 2009). LSM7 protein, whose mRNA levels were also increased in both DA agonist treatments (Popesku et al., 2010) also forms part of the spliceosome complex (Salgado-Garrido et al., 1999). The increase in both of these factors in response to either DA agonist suggests that blockage of either of these receptors would inhibit transcription of particular components of the spliceosome, and thus decrease splicing activity, thereby decreasing the amount of a particular splice variant. The observed decrease of the D2 short isoform splice variant in response to both D1 and D2 antagonists (Popesku et al., 2011b) supports this hypothesis.

Only three annotated genes/ESTs were identified in the telencephalon that were increased in response to D2 receptor agonists and decreased in response to D2 receptor blockage or DA depletion. This indicates that DA, acting through the D2 receptor, regulates these genes/ESTs. That relatively few genes affected by DA manipulation in the telencephalon was a surprising finding. While we expected tissue-specific responses to the various pharmacological treatments, we may have expected more than three genes to be affected in the Tel. In the case of D2 receptor, mRNA levels are high and specifically but widely expressed in regions of both Hyp and Tel of the African cichlid fish, *Astatotilapia burtoni* (O’Connell et al., 2011). However, it is not only the expression of receptors that will determine the response to an exogenous pharmacological agent, but also the ongoing effects of endogenous DA levels that are acting on both D1 and D2 receptors *in vivo*. It is clear in both goldfish and the cichlid, that DAergic innervation in the Hyp and Tel are extensive but clearly different, depending on the specific sub-region of each tissue (Hornby and Piekut, 1990; O’Connell et al., 2011). The clear difference in the global expression patterns in response to the various DA manipulations we report for goldfish Hyp and Tel supports this. Moreover, the type of cells expressing those receptors in each tissue will undoubtedly be different, so we do indeed expect major tissue differences.

Two of the DA-regulated genes/ESTs in the telencephalon are leucine-rich ppr-motif containing protein (LRPPRC) and solute carrier family 2 (facilitated glucose fructose transporter) member 5 (SLC2A5; glucose transporter 5; GLUT5). LRPPRC is a core nucleoid protein (Bogenhagen et al., 2008) and is hypothesized to have a regulatory role in the integration of the cytoskeleton with vesicular trafficking, nucleocytosolic shuttling, transcription, chromosome remodeling, and cytokinesis based on its interactions with other proteins by yeast 2-hybrid analysis (Liu and McKeehan, 2002). The third gene regulated by D2 in the telencephalon, CCAAT/enhancer-binding protein beta (C/EBPβ), is particularly interesting. CaMKII phosphorylates C/EBPβ (Wegner et al., 1992), which, in turn, activates transcription factor-1 (ATF1; Shimomura et al., 1996), among other things. Methamphetamine administration to mice caused a dose-dependent increase in ATF1 and CREB DNA-binding activities (Lee et al., 2002). As CaMKIIα protein levels were increased in response to DA agonists (Popesku et al., 2010), a working hypothesis of DAergic regulation of gene expression in the neuroendocrine brain of goldfish through the increase in ATF1 can thus be put forth.

Sub-network enrichment analysis takes advantage of previously characterized interactions between genes (expression relationships) and proteins (binding relationships). It is also able to associate genes and proteins with cell processes or diseases. The SNEA approach was developed by Ariadne (Pathway Studio^®^). Briefly, data on molecular interactions are retrieved from the ResNet nine database which is compiled using MedScan. The database contains over 20 million PubMed abstracts and approximately 900 K full-text articles (May 27, 2011). A background distribution of expression values in the gene list is calculated by an algorithm. This is followed by a statistical comparison between the sub-network and the background distribution using a Mann–Whitney *U*-Test, a *p*-value is generated that indicates the statistical significance of difference between two distributions (additional details can be found in the technical bulletin pg. 717 from Pathway Studio 7.0). SNEA has similar objectives to Ingenuity Pathway analysis and each is a useful tool to visualize molecular datasets. SNEA is different from KEGG which uses well defined biochemical and molecular pathways. SNEA has been applied in biomarker discovery in mammals (Kotelnikova et al., 2012) and for gene and protein networks in teleost fishes (Martyniuk et al., 2012; Trudeau et al., 2012). For this study, we chose to use Pathway Studios to visualize our data.

There were three major categories of the SNEA identified in the current study: cell signaling (STAT3, SP1, SMAD, Jun/Fos), immune response (IL-6, IL-1β, and TNF, cytokine, NF-κB), and cell proliferation and growth (IGF1, TGFβ1). Inflammatory pathways modulated by DA have been characterized in mouse models and have been associated with degenerative processes and cytokines released from glial cells play important roles in mediating cellular responses to injury due to neurotoxicants such as MPTP. For example, old male and female transgenic mice injected intraperitoneally with MPTP (15 mg/kg for 2 days at two injections/day) caused males to have dramatic increases in IL-1β luciferase reporter gene activity that correlated to the increased susceptibility of dopaminergic neurons to MPTP toxicity found in old male mice (Bian et al., 2009). In the same study, mRNA levels of TNF-α and IL-6 were not changed, but notable here is that genes affected downstream of IL-6 and TNF signaling were altered by DA in the goldfish hypothalamus, suggesting that these signaling cascades can be sensitive to dopaminergic inputs. In support of these data, both mRNA and protein levels for various cytokines (IL-1β, TNF-α, and IL-6) and expression of their receptors were significantly increased in the substantia nigra of MPTP-treated mice (Lofrumento et al., 2011). Here we identify putative gene targets and subsequent genomic effects that may occur after cytokine induction in the vertebrate CNS. A recent review by O’Callaghan et al. (2008) discuss the role of MPTP in inflammation in relation to cytokine signaling, including cytokines identified in the goldfish hypothalamus such as IL-1β and IL-6. Lastly, in regards to the inflammatory response in the goldfish, many of the cell signaling cascades are also involved in the immune response. For example, JAK/STAT3 signaling plays a role in inflammation in the mammalian brain in response to MPTP (Sriram et al., 2004). Therefore, the gene set node for cell signaling molecules (e.g., STAT) identified in the goldfish may directly stimulate inductions in cytokines.

Gene targets of IGF1 and TGFβ were also affected in expression after DA depletion and DA agonism. IGF1 activates RAS, P13K, and AKT signaling pathway to stimulate growth and differentiation of cells. TGF-β is a member of the transforming growth factor family that is involved in cell differentiation and regulation of the immune system. Both these signaling pathways are known to have a role in dopaminergic signaling and to be associated with the onset of neurodegenerative diseases. There are reports to suggest that IGF signaling may be involved in neuroprotection within the CNS. IGF1 has been shown to have protective role in MPP +  induced neurotoxicity in human neuroblastoma SH-EP1 cells by inhibiting apoptotic processes (Wang et al., 2010) and female rats treated with the neurotoxin 6-hydroxydopamine (6-OHDA) did not show reduced tyrosine hydroxylase immunoreactivity (a marker for DA toxicity) after intracerebroventricular infusion of IGF1 substantia nigra compared to those without the treatment (Quesada et al., 2008). The effect of IGF1 was dependent upon the PI3K/Akt pathway. It is plausible that gene expression changes in the goldfish hypothalamus in response to DA depletion and DA receptor activation are protective responses to DA-mediated neurotoxicity. Tong et al. (2009) investigated IGF distribution in human post-mortem brain tissues and report that IGF-I expression was significantly elevated in the frontal cortex of Parkinson’s patients while IGF-II expression was significantly reduced in the frontal white matter of PD patients. Thus, there are complex interactions between different IGF signaling pathways in the neurodegenerative brain (IGF1 and IGF2), however experimental evidence associates IGF in these processes. Similar to IGF1, TGFβ signaling targets are implemented in DA signaling in the goldfish hypothalamus. This pathway has also been implicated in neurodegeneration (Andrews et al., 2006) and the TGFβ signaling pathway can be modulated with DA treatments (Recouvreux et al., 2011).

Fish models are increasingly being used for investigations into the mechanisms of disease occurrence and progression (Weinreb and Youdim, 2007). Here we provide examples and demonstrate the usefulness of implementing SNEA to gain increased insight into key regulators underlying neurotransmitter signaling in the neuroendocrine brain and uncover novel associations between disease states and pharmacological treatments. In so doing, we provide a foundation for future work on dopaminergic regulation of gene expression in fish.

## Authors’ Contributions

Jason T. Popesku conceived of the study, designed and carried out the experiments, analyzed the data, and drafted the manuscript. Christopher J. Martyniuk participated in the design of the experiments, performed the sub-network enrichment analysis, and helped draft the manuscript. Vance L. Trudeau helped conceive the individual experiments, participated in the design and coordination of the study, and helped to draft the manuscript. All authors read and approved the final manuscript.

## Conflict of Interest Statement

The authors declare that the research was conducted in the absence of any commercial or financial relationships that could be construed as a potential conflict of interest.

## Appendix

**Table A1:** **ESTs were manually selected based on identical AURATUS GeneIDs and on the basis of differential regulation in opposite directions for MPTP or the antagonists vs. agonists, or in the same direction for MPTP vs. antagonists**.

Tissue	AURATUS ID	Best blast hit	DA depletion or receptor blockage			DA mimic		Accession
			MPTP + aMPT	SCH 23390	Sulpiride	SKF 38393	LY171555	
Hyp	08j13	14 kDa Apolipoprotein	−1.5			1.7		CF662566
Hyp	08b22	17-Beta hydroxysteroid dehydrogenase type 12B, 3-ketoacyl-CoA reductase type B	1.4			−1.7		CA968619
Hyp	16j14	26s Protease regulatory subunit 4	−1.4			1.4		CA966407
Hyp	08e14	40S Ribosomal protein S27	−1.5			1.7		CA968660
Hyp	07f01	Abhydrolase domain containing 12	−1.6			1.8		CA967283
Hyp	22n08	Adenylate kinase 3-like 1	1.3			−1.5		CA969490
Hyp	08k20	Aldehyde dehydrogenase 7 family, member A1	−1.3				1.3	CA968758
Hyp	03h23	Aldolase C	1.4			−1.6		DY231930
Hyp	05f06	Alpha-2-macroglobulin-1	−1.6			1.5	2.1	CF662428
Hyp	22i24	Alpha-actin	1.4			−1.5		CA969403
Hyp	09p02	Angiotensinogen	−1.5			1.8	1.3	CA964907
Hyp	09j02	Apolipoprotein a-iv	−1.5			1.7		CA966743
Hyp	16n14	Apolipoprotein e	−1.3			2.4		CF662778
Hyp	04a17	Aromatase b	1.3			−1.7		FG392770
Hyp	14k14	arp2 Actin-related protein 2 homolog	−1.3			2.3	1.3	CA964468
Hyp	12l13	asf1 Anti-silencing function 1 homolog b (*cerevisiae*)	−1.3			1.9		CA966040
Hyp	16l15	atp-Binding sub-family f member 2	−1.3			1.6		CA966450
Hyp	16o14	BC-10 protein	−1.3			1.9		CA966992
Hyp	03o22	Beta-actin	1.3			−1.6		DY232011
Hyp	22l24	Branched chain ketoacid dehydrogenase kinase		1.6		−1.8		CA969461
Hyp	02a23	Calmodulin 1b	1.2			−1.7		FG392553
Hyp	17j08	*Carassius auratus* mRNA for BC-10 protein	1.3			−1.5		CA966515
Hyp	10f12	Carp DNA sequence from clone carpf-118, complete sequence	1.5			−1.6		CA964207
Hyp	16e02	Chromosome 9 open reading frame 82	−1.4			1.6		CA966153
Hyp	14g01	Claudin 23	−1.4			1.8		CA964745
Hyp	14k02	Coiled-coil domain containing 47	−1.3			2.1		CA964457
Hyp	19a04	Cold shock domain-containing protein e1		−1.4		1.5	1.3	CA964993
Hyp	08o15	Complement C3-H2	−1.4			1.6		CA970421
Hyp	08b20	Complement component q subcomponent-like 4	−1.3				1.3	CA968617
Hyp	02c23	Creatine kinase b variant 1	1.3			−1.6		DY231608
Hyp	02n10	Creatine testis isozyme	1.2			−1.5		DY231690
Hyp	21l19	C-type lectin	1.5	−1.7		−1.7		CA969207
Hyp	19a14	Cubilin (intrinsic factor-cobalamin receptor)	−1.4			1.4		CA964997
Hyp	17g09	cxxc Finger 1 (phd domain)	1.3			−1.7		CA964951
Hyp	11i01	*Cyprinus carpio* DN1 mRNA for DNase I, complete cds	−1.4			1.7		CA965953
Hyp	06d13	Cytochrome p450 like	−1.4				1.6	CA965416
Hyp	05l01	Cytokine induced apoptosis inhibitor 1	−1.4			2.3		CA966987
Hyp	10g12	*Danio rerio* HECT domain containing 1 (hectd1), mRNA	1.5			−1.5		CA967652
Hyp	19h04	*Danio rerio* heterogeneous nuclear ribonucleoprotein A/B, mRNA (cDNA clone MGC:55953), complete cds		−1.4		1.8		CA965823
Hyp	24j13	*Danio rerio* lin-7 homolog A (*C. elegans*; lin7a), mRNA	−1.4				1.4	CA969901
Hyp	07m24	*Danio rerio* non-metastatic cells 4, protein expressed in (nme4), mRNA	1.4			−1.7		CA964093
Hyp	22c24	*Danio rerio* SET translocation (myeloid leukemia-associated) A (seta), mRNA	1.3			−1.6		CA969283
Hyp	08g15	*Danio rerio* zgc:110605 (zgc:110605), mRNA	−1.3			1.6		CA970392
Hyp	12e04	*Danio rerio* zgc:55886 (zgc:55886), mRNA	−1.3			1.7	1.3	CA966744
Hyp	24k14	*Danio rerio* zgc:77060 (zgc:77060), mRNA		1.9		−1.7		CA969922
Hyp	22m10	*Danio rerio* zgc:92169 (zgc:92169), mRNA	1.3			−1.6		CA969469
Hyp	09b02	*Danio rerio* zgc:92371 (zgc:92371), mRNA	−1.4			2.0	1.3	CA964765
Hyp	03j12	*Danio rerio* neuron-specific protein family member 1 (brain neuron cytoplasmic protein 1) mRNA	1.3			−1.6		FG392599
Hyp	03f23	Deoxyribonuclease I-like 3	1.5			−1.5		DY231911
Hyp	23k24	e3 Ubiquitin protein ligase	1.6			−1.6		CA968074
Hyp	02i24	Ependymin	1.3			−1.6		DY231713
Hyp	03o21	Ependymin	1.4			−1.7		DY232010
Hyp	24a12	eph Receptor a7	1.6			−2.1		CA969719
Hyp	15a10	Equilibrative nucleoside transporter 1	1.3			−1.6		CA965545
Hyp	07b01	Eukaryotic translation elongation factor-1 gamma	−1.5			1.7		CA966738
Hyp	20j14	Eukaryotic translation initiation factor 2, subunit 1 alpha	−1.3	−2.0		2.3		CA966561
Hyp	09e01	Fibronectin 1b	−1.3			2.0	1.3	CA964120
Hyp	24j21	fk506-Binding protein 1a	1.3			−1.5		CA966789
Hyp	03o09	Fructose-bisphosphate aldolase c	1.4			−1.6		FG392624
Hyp	10m11	g Protein-coupled family group member c	1.3			−1.6		CA967701
Hyp	17n11	Gamma-glutamyl cyclotransferase	1.3			−1.7		CA965786
Hyp	02g12	*Gasterosteus aculeatus* clone cnb214-a06 mRNA sequence	1.3			−1.5		DY231579
Hyp	03i20	Glutamine synthetase	1.2			−1.5		DY231974
Hyp	10d04	Glutathione peroxidase 3	1.4			−1.5		CA964192
Hyp	23o12	Glyceraldehyde 3-phosphate dehydrogenase	2.0			−2.1		CA968103
Hyp	08h01	Glyceronephosphate-*O*-acyltransferase	−1.6			2.2		CA968696
Hyp	14b13	Granulin 1	−1.3			1.5		CA964295
Hyp	19m14	h2a Histone member y2	−1.4			1.6		CA965061
Hyp	14k03	Heat shock protein 90 beta	−1.3			1.7		CA964458
Hyp	14i04	HECT domain containing 1		−1.4		1.5		CA964417
Hyp	24o12	Hexokinase I	1.6			−1.9		CA969997
Hyp	08g14	High-density lipoprotein binding protein	−1.4			1.6		CA968690
Hyp	19d02	Hydroxysteroid (17-beta) dehydrogenase 10	−1.3			2.2	1.3	CA965806
Hyp	03i10	Immunoglobulin mu heavy chain	1.5			−1.5		FG392590
Hyp	04j23	Jumonji domain containing 3	1.3			−1.5		FG392963
Hyp	13o14	Latexin		−1.7		1.6		CF662717
Hyp	22g07	Leucine-rich repeat (in flii) interacting protein 1	1.2			−1.7		CA969350
Hyp	11p01	Leucine-rich repeat containing 58	−1.3			2.2		CF662658
Hyp	19f13	loc548392 Protein	−1.4			2.0		CA969104
Hyp	14m01	Malate dehydrogenase 1, NAD (soluble)	−1.3			1.8	1.3	CA964750
Hyp	12k14	Male-specific protein	−1.3			1.9		CA970272
Hyp	22o11	Map microtubule affinity-regulating kinase 4	1.5			−2.0		CA969512
Hyp	21l16	Membrane palmitoylated	1.9			−1.6	1.3	CA966525
Hyp	09p22	Methylcrotonoyl-coenzyme a carboxylase 2	1.5			−1.8		CA964915
Hyp	22k08	MHC class I antigen	1.4			−2.0		CA969424
Hyp	08a03	mid1 Interacting g12-like protein	−1.3			1.6		CA970376
Hyp	09k02	mid1 Interacting g12-like protein	−1.4			1.7		CA964854
Hyp	08l01	Middle subunit	−1.4			2.5		CA965449
Hyp	03k10	Midkine-related growth factor b	1.4			−1.5		FG392604
Hyp	12n01	Mitochondrial ribosomal protein l19	−1.6			1.5		CA966046
Hyp	19p16	Mitochondrial ribosomal protein l20		−1.4		2.0		CA967272
Hyp	11j11	Mitogen-activated protein kinase 7 interacting protein 3	1.4			−1.7		CF662634
Hyp	12p13	m-Phase phosphoprotein 6	−1.5			2.1		CA966058
Hyp	06g06	Myelocytomatosis oncogene b	1.3			−2.7		CF662485
Hyp	14n02	Myosin regulatory light chain	−1.3			1.6		CA964520
Hyp	24b19	nck Adaptor protein 2	1.4			−1.5		CA969746
Hyp	19l18	Negative elongation factor d		−1.8		1.5		CA965844
Hyp	03i12	Nel-like protein 2	1.3			−1.7		FG392591
Hyp	16k15	nlr Card domain containing 3		−1.3		1.8		CF662774
Hyp	18c18	nol1 nop2 Sun domain member 2		−1.5		−1.5		CA964613
Hyp	08o01	Novel protein	−1.4			1.5		CA968809
Hyp	11d07	Novel protein	1.3			−1.5		CF662614
Hyp	15i06	Novel protein (zgc:136439)		−1.6		1.6		CA965636
Hyp	15b13	Novel protein lim domain only 3 (rhombotin-like 2) zgc:110149)	−1.4			2.0		CA965552
Hyp	11e15	Novel sulfotransferase family protein	−1.3			1.9		CA965939
Hyp	19e01	Nuclear receptor sub-family group member 2	−1.4			2.1		CA966183
Hyp	15e23	Phosducin-like 3		1.5		−1.6		CA966723
Hyp	12l11	Plasma retinol-binding protein 1	1.3			−1.5		CA966039
Hyp	03k09	Poplar cDNA sequences	1.3			−1.5		FG392603
Hyp	12o13	PREDICTED: *Danio rerio* hypothetical LOC560379 (LOC560379), mRNA	−1.3			1.5		CA966719
Hyp	24d23	PREDICTED: *Danio rerio* hypothetical LOC567058 (LOC567058), mRNA	1.3			−1.5		CA966814
Hyp	15i02	PREDICTED: *Danio rerio* hypothetical protein LOC553758 (LOC553758), mRNA	−1.3			1.6		CA965635
Hyp	19f14	PREDICTED: *Danio rerio* hypothetical protein LOC792300 (LOC792300), mRNA		−1.3		2.5		CA965818
Hyp	07i10	PREDICTED: *Danio rerio* im:7148349 (im:7148349), misc RNA	1.7			−1.6		CA964049
Hyp	08j02	PREDICTED: *Danio rerio* similar to Chromosome 19 open reading frame 43, transcript variant 1 (LOC560758), mRNA	−1.5			2.1		CA968727
Hyp	19o03	PREDICTED: *Danio rerio* similar to dipeptidyl-peptidase 6, transcript variant 1 (LOC566832), mRNA		−1.6		1.4		CA966233
Hyp	12o22	PREDICTED: *Danio rerio* similar to histocompatibility 28 (LOC555357), mRNA		−1.6		1.5		CA970293
Hyp	17g21	PREDICTED: H3 histone, family 3B			−1.3		1.8	CA964956
Hyp	15o14	PREDICTED: hypothetical protein [*Danio rerio*]	−1.2			2.0		CA965715
Hyp	22g11	PREDICTED: hypothetical protein LOC337077, partial [*Danio rerio*]	1.3			−2.0		CA969354
Hyp	08g04	Prostaglandin h2 d-isomerase	−1.5			1.5		CA968684
Hyp	22p03	Proteasome (macropain) 26s non- 4	1.4			−1.6		CA969527
Hyp	12b01	Proteasome (macropain) alpha 5	−1.5			2.0	1.3	CA965983
Hyp	12i01	Purine nucleoside phosphorylase	−1.5			1.6		CA967769
Hyp	22b23	Response gene to complement 32	1.3			−2.0		CA969259
Hyp	22g21	Ribosomal protein l13	1.5			−1.7		CA969362
Hyp	08o16	Ribosomal protein l27a	−1.3			1.5		CA968817
Hyp	12d13	Ribosomal protein l27a	−1.5			1.6		CA965998
Hyp	09o01	Serine incorporator 1	−1.4				1.5	CA964172
Hyp	04c11	Sesbania drummondii clone ssh-36_01_a09_t3 mRNA sequence	1.2			−1.5		FG392711
Hyp	21a01	sh3-Domain grb2-like 2	−1.5			1.7	1.3	CA967895
Hyp	09g14	si:ch211-Protein	−1.4			1.8		CA964823
Hyp	11l01	*Siniperca chuatsi* 28S ribosomal RNA gene, partial sequence	−1.5			1.8		CA966341
Hyp	24i19	StAR-related lipid transfer (START) domain containing 4	1.4			−2.1	1.6	CA969885
Hyp	09n02	Sterol-c5-desaturase (fungal delta-5-desaturase) homolog (*cerevisiae*)	−1.3			2.5		CA964885
Hyp	12p21	Surfeit 4		−1.5		1.6		CA966062
Hyp	20o02	Tetraspanin 9	−1.6			1.6		CA965906
Hyp	24i22	Transaldolase 1	1.4			−1.6		CA969888
Hyp	15f10	Translocon-associated protein subunit delta precursor	1.3			−1.8		CA965601
Hyp	12f01	Transthyretin precursor	−1.3			3.0		CA966004
Hyp	07h01	Triosephosphate isomerase	−1.3			1.8		CA968504
Hyp	14f24	Troponin c-type 2	1.4			−2.1		CA964383
Hyp	21g17	Troponin c-type 2		−1.5		1.6		CA967929
Hyp	22g09	Tubulin alpha 8 like 4	1.3			−1.8		CA969352
Hyp	03o23	Tubulin beta-2c	1.4			−1.5		FG392672
Hyp	17j23	Tubulin beta-2c chain	1.4			−1.5		CA965774
Hyp	14f02	u2 Small nuclear RNA auxiliary factor-1		−1.7		2.4	1.3	CA964363
Hyp	22l09	Vacuolar protein sorting 13c	1.3			−1.5		CA969449
Hyp	20j02	Vacuolar protein sorting 4a	−1.5			1.7	1.6	CA966560
Hyp	14j12	Vimentin	1.4			−1.5		CA964445
Hyp	24i24	Vimentin	1.4			−1.7		CA969890
Hyp	12i13	Vitellogenin 2	−1.3			1.4		CA967775
Hyp	03a21	Zebrafish DNA sequence from clone ch1073-368i11 in linkage group complete sequence	1.4			−2.0		DY231868
Hyp	16n18	Zebrafish DNA sequence from clone CH211-11J2 in linkage group 7, complete sequence		−1.3		1.8		CA966457
Hyp	19e02	Zebrafish DNA sequence from clone CH211-126C2 in linkage group 14, complete sequence	−1.4			2.0		CA965014
Hyp	24p11	Zebrafish DNA sequence from clone CH211-128E9 in linkage group 15, complete sequence	1.4	−1.6		−1.8		CA970016
Hyp	03f11	Zebrafish DNA sequence from clone ch211-132l2 in linkage group complete sequence	1.4			−1.7		DY231843
Hyp	16n17	Zebrafish DNA sequence from clone CH211-134D6, complete sequence		−1.3		1.4		CF662780
Hyp	09m02	Zebrafish DNA sequence from clone CH211-157C7 in linkage group 7, complete sequence	−1.4			1.7		CA964874
Hyp	03p21	Zebrafish dna sequence from clone ch211-194m7 in linkage group 25 contains the gene for a novel proteinvertebrate ndrg family member 4 and a complete sequence	1.3			−1.5		DY232016
Hyp	19e13	Zebrafish DNA sequence from clone CH211-221E5 in linkage group 8, complete sequence	−1.3			1.5		CA966187
Hyp	02c11	Zebrafish DNA sequence from clone ch211-271d10 in linkage group complete sequence	1.4			−1.5		DY231543
Hyp	24d22	Zebrafish DNA sequence from clone CH211-286F18 in linkage group 14, complete sequence	1.4			−1.5		CA969787
Hyp	22o22	Zebrafish DNA sequence from clone CH211-63O20 in linkage group 20, complete sequence	1.3			−1.5		CA969522
Hyp	24h12	Zebrafish DNA sequence from clone CH211-65M8, complete sequence	1.5			−1.6		CA969857
Hyp	22j22	Zebrafish DNA sequence from clone DKEY-106L3 in linkage group 10, complete sequence	1.3			−1.6		CA969418
Hyp	19m15	Zebrafish DNA sequence from clone DKEY-10B15 in linkage group 10, complete sequence	−1.4	−1.5		1.8		CA966228
Hyp	03h21	Zebrafish DNA sequence from clone dkey-13a3 in linkage group complete sequence	1.4			−1.5		DY231928
Hyp	22f11	Zebrafish DNA sequence from clone DKEY-14A21 in linkage group 12, complete sequence	1.4	−1.7		−1.6		CA969332
Hyp	24h11	Zebrafish DNA sequence from clone DKEY-180P18 in linkage group 4, complete sequence	1.8			−1.9		CA969856
Hyp	14g09	Zebrafish DNA sequence from clone DKEY-210O7 in linkage group 6, complete sequence	1.5			−1.4		CA967264
Hyp	22k16	Zebrafish DNA sequence from clone DKEY-216E24 in linkage group 9, complete sequence	1.3			−1.4		CA969432
Hyp	13k21	Zebrafish DNA sequence from clone DKEY-228N9 in linkage group 11, complete sequence	1.3			−1.6		CA967861
Hyp	24j22	Zebrafish DNA sequence from clone DKEY-231K15 in linkage group 3, complete sequence	1.3			−1.6		CA969909
Hyp	15f02	Zebrafish DNA sequence from clone DKEY-242H9 in linkage group 18, complete sequence	−1.4			1.6	1.3	CA965596
Hyp	03i22	Zebrafish DNA sequence from clone dkey-266h7 in linkage group 5 contains the 3 end of the gene for a novel proteinvertebrate mitochondrial ribosomal protein l41the gene for a novel proteinvertebrate patatin-like phospholipase domain containing 6the gene for a novel protein and the 3 end of the gene for a novel proteinvertebrate atp-binding cassette sub-family a abc1 member 2 complete sequence	1.3			−1.7		FG392637
Hyp	18b02	Zebrafish DNA sequence from clone DKEY-3P10 in linkage group 23, complete sequence		−1.6		1.6		CA968927
Hyp	23k09	Zebrafish DNA sequence from clone DKEY-40M6 in linkage group 16, complete sequence	1.3			−1.5		CF662916
Hyp	10m12	Zebrafish DNA sequence from clone DKEYP-1H4 in linkage group 18, complete sequence	1.4			−1.6		CA967702
Hyp	22p07	Zebrafish DNA sequence from clone DKEYP-64A3 in linkage group 2, complete sequence	−1.4				1.3	CA969530
Hyp	19o08	Zinc and double phd fingers family 2		−1.5		1.5		CA965067
Hyp	23a24	Zinc finger ccch-type containing 7a	2.0			−2.3		CA967982
Hyp	15i14	Zinc finger protein 782	−1.3			2.0		CA965639
Hyp	20c13	Zona pellucida glycoprotein		−1.6		1.7		CA966260
Tel	12o17	ccaat Enhancer-binding protein beta			−1.6		1.7	CA967804
Tel	12e10	Leucine-rich ppr-motif containing	−1.8				1.6	CA970240
Tel	14f04	Solute carrier family 2 (facilitated glucose fructose transporter) member 5			−1.3		1.9	CA964365

*All ESTs were identified as being statistically significantly differentially regulated (*q* < 5%) in all treatments. Only those with BLAST hits (NCBI), obtained with Blast2GO, are shown. In the case where a suitable BlastX hit was unavailable, the best BlastN hit is used. Duplicate names may exist in the list, but were not identified by sequence overlap (cap3) and may represent separate genes or individual isoforms. The median “minimum ExpectValue” = 1.9E−57 and the average “mean similarity” = 84.8 ± 1%*.
